# Comparing the Sedative Effect of Dexmedetomidine and Propofol in Mechanically Ventilated Patients Using Salivary Alpha-Amylase as a Stress Marker: A Randomized Open-Label Trial

**DOI:** 10.5812/aapm-149364

**Published:** 2024-10-27

**Authors:** Ahmed Mohamed Ibrahim, Mohammed Said ElSharkawy, Reda Khalil Abdelrahman, Abdallah Elabd Hassan, Mohammed Gaber Ibrahim Saad, Ismail Ahmed Elzoughari, Abdelkarem Hussini Ismail Alsayed, Asmaa Abdelbadie, Rehab Abd El Fattah Helal

**Affiliations:** 1 Anesthesiology, Surgical Intensive Care and Pain Medicine Department, Faculty of Medicine, Tanta University, Tanta, Egypt; 2 Anesthesiology, Intensive Care and Pain Management Department, Faculty of Medicine, Al-Azhar University, Damietta, Egypt; 3 King Abdulaziz University Hospital, Jeddah, Saudi Arabia; 4 Anesthesiology, Intensive Care and Pain Management Department, Faculty of Medicine, Al-Azhar University, Cairo, Egypt; 5 Clinical Pharmacy Department, Faculty of Pharmacy, Nahda University, Bani Suef, Egypt

**Keywords:** Dexmedetomidine, Propofol, Mechanical Ventilation, Stress, Salivary Alpha-Amylase

## Abstract

**Background:**

Patients receiving mechanical ventilation (MV) in the intensive care unit (ICU) experience significant distress, which triggers a stress response.

**Objectives:**

This study aimed to assess the effectiveness of dexmedetomidine and propofol in reducing stress levels, using salivary alpha-amylase (SAA) as a specific indicator of stress.

**Methods:**

A randomized, open-label trial was conducted involving 40 patients newly placed on MV. In a parallel study design, participants were randomly assigned into two equal groups (n = 20) through the sealed envelope method using computer-generated randomization. Group D received dexmedetomidine at a dosage of 0.2 to 1.4 μg/kg/h, while group P received propofol at a dosage of 0.3 to 4 mg/kg/h for sedation. Salivary alpha-amylase levels were measured according to the kit manufacturer's protocol.

**Results:**

Salivary alpha-amylase levels were significantly lower in group D than in group P at 12, 24, 36, and 48 hours after the initiation of MV (P < 0.05). Heart rate and mean arterial pressure were also significantly lower in group D at 12, 18, and 24 hours (P < 0.05). The duration of MV was significantly shorter in group D compared to group P (4.4 ± 1.85 vs 6.1 ± 2.45 days, P = 0.018). There were no significant differences in ICU length of stay, mortality, or adverse events between the groups.

**Conclusions:**

Dexmedetomidine demonstrated superior stress-reducing effects compared to propofol in MV patients, as evidenced by lower SAA levels and improved hemodynamic stability. The shorter duration of MV in the dexmedetomidine group further suggests potential clinical benefits of its use in managing stress in MV patients.

## 1. Background

Critically ill patients on mechanical ventilation (MV) in the intensive care unit (ICU) often endure significant psychological distress, including feelings of despair, anxiety, heightened agitation, and increased strain (1, 2). One of the primary reasons for administering sedation to these patients is to prevent both involuntary and voluntary resistance to the endotracheal tube, which can lead to tachycardia, tachypnea, and elevated physiological stress markers (3). During the stress response, a variety of hormones are released, including epinephrine, cortisol, cytokines, growth factors, and activated components of the complement system (4).

The stress response can be alleviated by administering sedatives like alpha-2 receptor agonists, such as propofol or dexmedetomidine (5, 6). Sedation in patients requiring MV is commonly used to promote comfort, reduce pain and anxiety, and facilitate nursing care (7-9).

Propofol, a sedative-hypnotic anesthetic, is frequently used to induce sedation (10, 11). However, it has notable drawbacks, including a narrow therapeutic range and adverse effects such as hypotension, respiratory depression, hemodynamic instability, and infusion discomfort (12).

Dexmedetomidine, an alpha-2 adrenergic receptor agonist, induces sedation in MV patients, reduces delirium incidence, and has analgesic properties (13, 14). It has shown efficacy in enhancing patient comfort during MV, while maintaining a strong safety profile and reducing extubation times (15).

Salivary alpha-amylase (SAA) is an enzyme responsible for the hydrolysis of carbohydrates. It is synthesized by highly differentiated epithelial acinar cells in the exocrine salivary glands, primarily produced in the parotid glands (16). Multiple studies have provided empirical evidence supporting the reliability and validity of SAA activity as a biomarker for sympathetic activation during stress (17-20).

We hypothesized that dexmedetomidine would be more effective than propofol in reducing stress levels in mechanically ventilated patients, as measured by SAA levels, and would result in improved clinical outcomes, including a shorter duration of MV. 

## 2. Objectives

Therefore, this study aimed to assess the efficacy of dexmedetomidine versus propofol in reducing stress levels in MV patients, using SAA as a marker of stress.

## 3. Methods

This randomized open-label trial was conducted on 40 patients, aged 18 - 65 years of both sexes, who were newly placed on MV in the ICU at Tanta University Hospitals, Egypt, between October 2023 and April 2024. Patients were equally assigned (n = 20) to two groups: Group D received dexmedetomidine, while group P received propofol. The study was approved by the institutional ethical committee (ID: 36264PR353/9/23) and registered on clinicaltrials.gov (ID: NCT06098209). After a detailed explanation of the study's objectives, informed written consent was obtained from the patients' relatives.

Exclusion criteria included patients using inhaled steroids, medications known to affect salivary glands (such as antihypertensives, antidepressants, or antipsychotics) to minimize confounding factors in SAA measurement, a history of smoking or alcohol consumption due to potential alterations in salivary composition and flow rate, pregnant individuals, those on oral contraceptives due to ethical considerations and potential hormonal influences on SAA, patients with hypersensitivity to the research medications, and those undergoing adrenoreceptor agonist or antagonist treatment or menstruating.

### 3.1. Randomization and Blindness

In a parallel design, 40 patients were enrolled and randomly divided equally into two groups (20 each) using the sealed envelope method with computer-generated randomization. Group D received dexmedetomidine at varying doses ranging from 0.2 to 1.4 μg/kg/h, while group P received propofol at doses ranging from 0.3 to 4 mg/kg/h.

The open-label design was chosen due to the distinct visual differences between the medications (dexmedetomidine vs. propofol), making blinding of participants or researchers impractical. Upon admission to the ICU, medical history, clinical examinations, and routine laboratory investigations were conducted, and the causes for ICU admission were prospectively collected for all enrolled patients.

The trial was terminated if the patient exhibited persistent bradycardia [defined as a heart rate (HR) < 60 bpm] (21), newly developed second- or third-degree heart block, severe allergic reactions, suspected propofol-related infusion syndrome (characterized by refractory shock, rhabdomyolysis, acidosis, and kidney failure associated with high propofol exposure), or any significant adverse event linked to the treatment.

Heart rate and mean arterial blood pressure (MAP) were measured immediately after the initiation of MV, and then at 6, 30, 36, 42, and 48 hours between both groups.

The drugs were administered for 2 days with the goal of maintaining the Richmond Agitation-Sedation Scale (RASS) score between -3 to -2 for both groups. The RASS is used to assess sedation levels in ICU patients, consisting of ten points that range from agitation to deep sedation. The scale includes four points for agitation, from +1 (restlessness) to +4 (combativeness), one point for calmness and alertness (0), and five points for sedation, from -1 (drowsy) to -5 (unarousable). Each point is clearly defined to standardize the evaluation of agitation or sedation.

Saliva samples were collected immediately after MV was initiated and then every 12 hours for 2 days. A specially trained nurse placed a swab into each patient's mouth for 2 - 5 minutes to collect the samples. Salivary alpha-amylase concentrations were measured using commercially available ELISA kits, following the manufacturer’s instructions (Salimetrics, USA). The method employed a chromogenic substrate, 2-chloro-p-nitrophenol, linked to maltotriose, which α-amylase cleaves to produce 2-chloro-p-nitrophenol. This product was measured spectrophotometrically at 405 nm. After collection, saliva samples were refrigerated within 30 minutes and frozen at -20°C within 4 hours. Samples were then centrifuged at 1500 x g for 15 minutes, diluted at a 1:200 ratio with assay diluent, and 8 μL of each sample or control was added to wells, followed by 320 μL of pre-heated (37°C) α-amylase substrate. Optical density was measured at 1 minute and 3 minutes post-incubation.

The primary outcome of the study was the SAA level, while secondary outcomes included hemodynamic measurements, duration of MV, ICU length of stay, and adverse side effects.

### 3.2. Size of Sample Calculation

The sample size calculation was performed using G*Power 3.1.9.2 (Universitat Kiel, Germany). Based on a pilot study involving five patients in each group, the mean (± SD) SAA levels were 64.66 ± 11.66 U/mL in group D and 75.64 ± 7.36 U/mL in group P. Using an effect size of 1.126, a 95% confidence interval, and a power of 95%, with a group ratio of 1:1, the sample size was calculated. To account for potential dropouts, two additional cases were added to each group, resulting in a total recruitment of 20 patients per group.

### 3.3. Statistical Analysis

SPSS v27 (IBM^©^, Armonk, NY, USA) was used for statistical analysis. The normality of the data was assessed using the Shapiro-Wilks test and visual inspection of histograms. Quantitative parametric data were presented as mean ± standard deviation (SD) and analyzed using an unpaired Student’s *t*-test. For quantitative non-parametric data, the median and interquartile range (IQR) were presented and analyzed using the Mann-Whitney U test. Qualitative variables were displayed as frequency and percentage (%), and their association was evaluated using the chi-square test or Fisher’s exact test, as appropriate. A two-tailed P-value of < 0.05 was considered statistically significant.

## 4. Results

**Figure 1.**
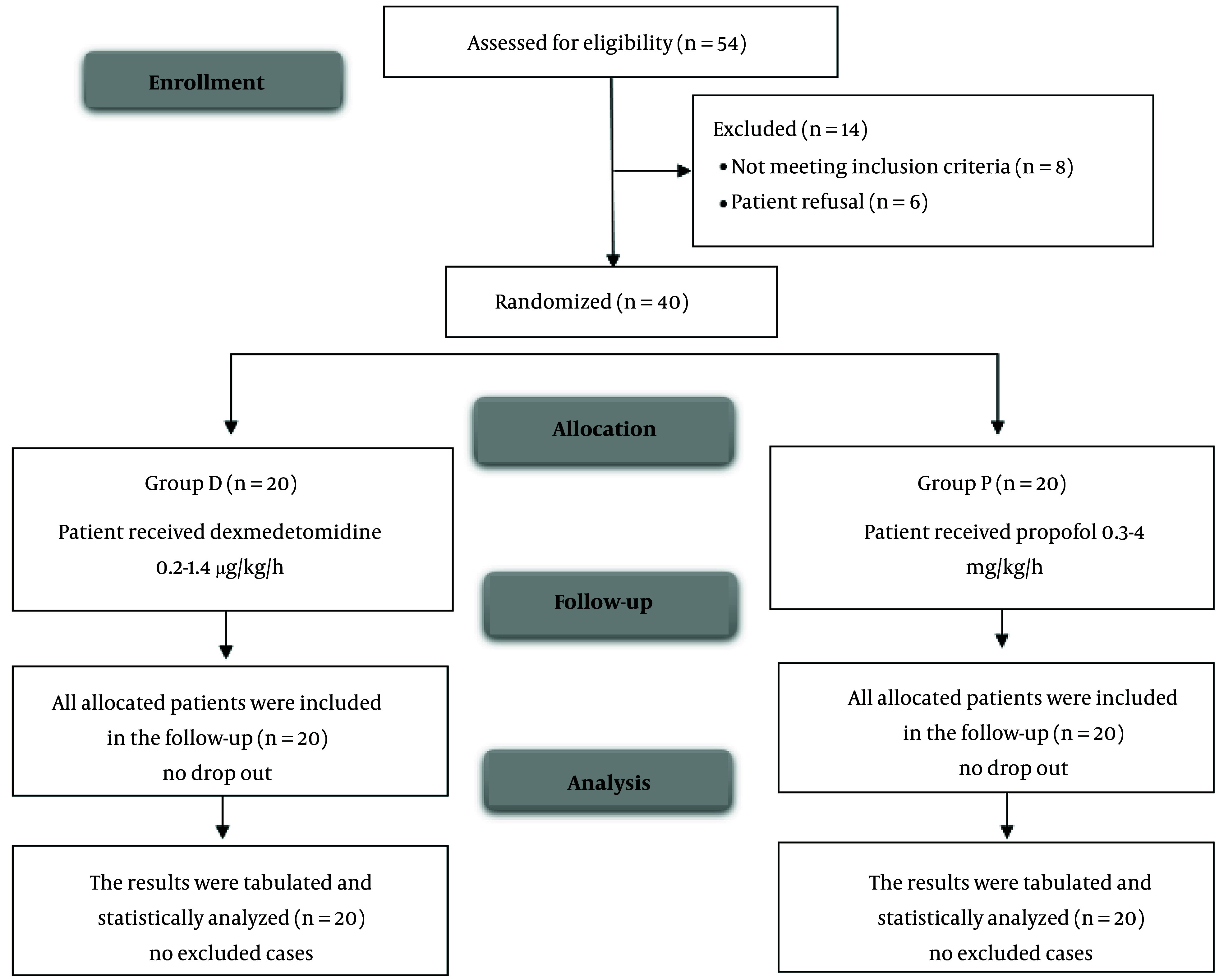
CONSORT flowchart of the enrolled patients

A total of 54 individuals were assessed for eligibility in this research, with eight patients not meeting the eligibility criteria and six patients declining to participate. The remaining patients were randomly assigned to two groups, with 20 patients in each group. All allocated patients were followed up and included in the statistical analysis (Figure 1). 

Demographic data, comorbidities, APACHE score, RASS score, and the causes of MV showed no significant differences between the two groups (Table 1). No significant difference in SAA levels was observed immediately after MV between the groups. However, SAA was significantly lower at 12h, 24h, 36h, and 48h in group D compared to group P (P < 0.05) (Table 2). 

**Table 1.** Demographic Data, Comorbidities, Acute Physiology and Chronic Health Evaluation Score, Richmond Agitation-Sedation Scale and Causes of Mechanical Ventilation of the Studied Groups ^a^

Variables	Group D; (n = 20)	Group P; (n = 20)	P-Value
**Age (y)**	42.9 ± 12.25	38.2 ± 12.45	0.236
**Gender**			0.519
Male	13 (65)	11 (55)	
Female	7 (35)	9 (45)	
**Weight (kg)**	76.75 ± 12.09	71 ± 13.11	0.157
**Height**	167.2 ± 7.59	166.15 ± 5.74	0.624
**BMI (kg/m** ^**2**^ **)**	27.53 ± 4.27	25.75 ± 4.58	0.212
**Comorbidities**			
Hypertension	8 (40)	6 (30)	0.507
Diabetes mellitus	5 (25)	3 (15)	0.694
Myocardial infarction	1 (5)	2 (10)	1
Chronic obstructive pulmonary disease	4 (20)	3 (15)	1
Coronary artery disease	1 (5)	1 (5)	1
**Causes of MV**			0.833
Septic shock	3 (15)	4 (20)	
Pneumonia	6 (30)	8 (40)	
Traumatic brain injury	3 (15)	4 (20)	
Acute respiratory distress syndrome	6 (30)	4 (20)	
**APACHI score**	19.65 ± 7.2	16.55 ± 6.03	0.148

Abbreviations: BMI, Body Mass Index; APACHI, acute physiology and chronic health evaluation; MV, mechanical ventilation.

^a^ Values are expressed as No. (%) or mean ± SD.

**Table 2.** Salivary Alpha-Amylase of the Studied Groups ^a^

Variables	Group D; (n = 20)	Group P; (n = 20)	P-Value
**Immediately after MV**	69.78 ± 7.38	72.89 ± 5.98	0.151
**12h**	66.88 ± 8.9	72.14 ± 6.62	0.041 ^b^
**24h**	63.8 ± 7.07	68.36 ± 5.99	0.034 ^b^
**36h**	59.36 ± 7.35	66.29 ± 6.03	0.002 ^b^
**48h**	52.41 ± 7.33	61.48 ± 6.19	< 0.001 ^b^

Abbreviation: MV, mechanical ventilation.

^a^ Values are expressed as mean ± SD.

^b^ P < 0.05 was considered statistically significant.

**Figure 2.**
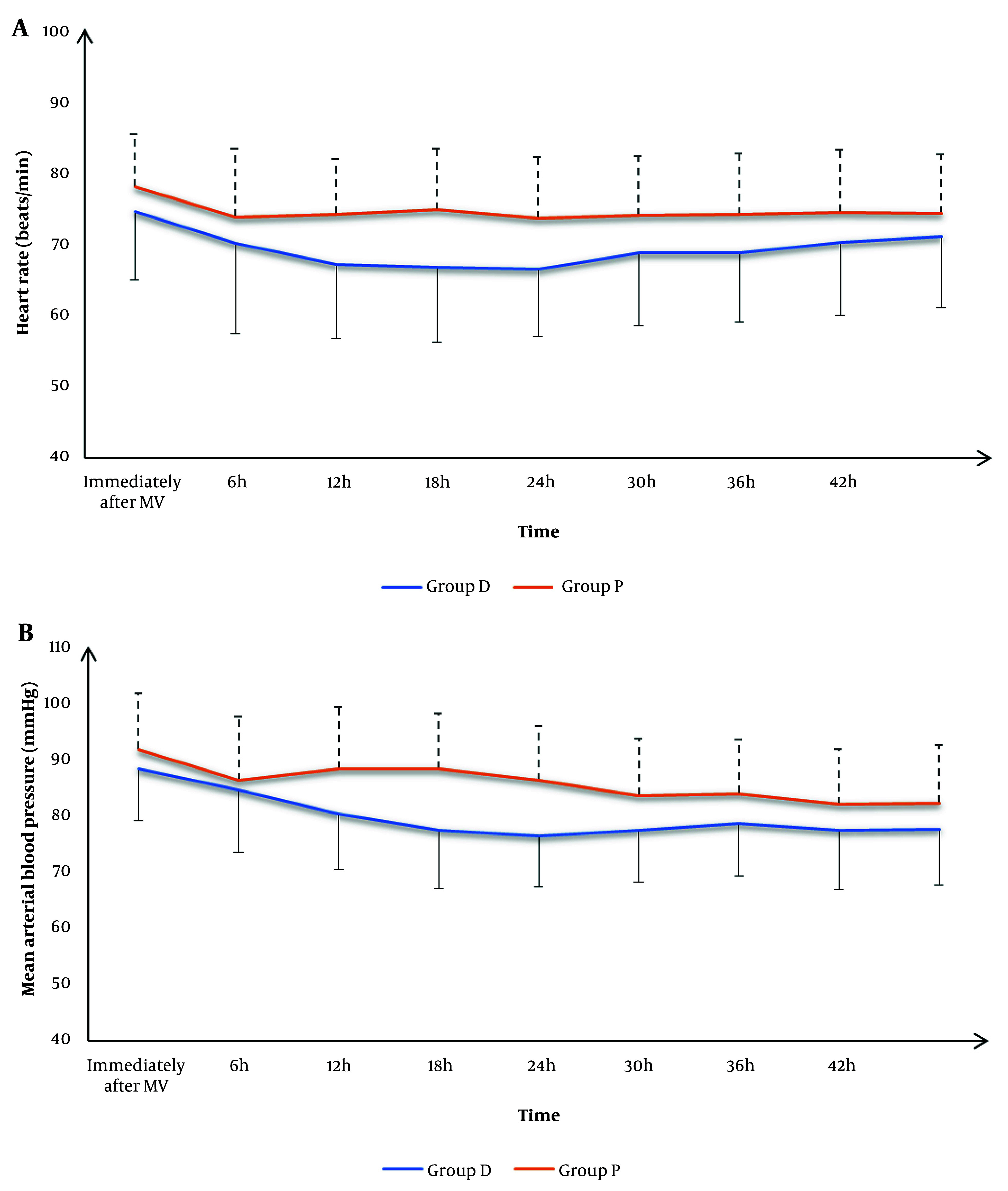
A, heart rate (HR); and B, mean arterial pressure (MAP) of the studied groups

There was no significant difference in HR and MAP immediately after MV, or at 6h, 30h, 36h, 42h, and 48h between the two groups. However, group D exhibited significantly lower HR and MAP at 12h, 18h, and 24h compared to group P (P < 0.05) (Figure 2). 

The duration of MV was significantly shorter in group D compared to group P (P = 0.018). No significant differences were observed in ICU length of stay, mortality, bradycardia, or hypotension between the two groups (Table 3). 

**Table 3.** Mechanical Ventilation, Intensive Care Unit Length of Stay, ICU Mortality and Complication of Studied Groups ^a^

Variables	Group D; (n = 20)	Group P; (n = 20)	P-Value
**Duration of MV (days)**	4.4 ± 1.85	6.1 ± 2.45	0.018 ^b^
**ICU length of stay (days)**	6.85 ± 1.27	7.8 ± 1.94	0.074
**ICU mortality**	2 (10)	4 (20)	0.661
**Complications**			
**Bradycardia**	4 (20)	2 (10)	0.661
**Hypotension**	7 (35)	4 (20)	0.480

Abbreviations: MV, mechanical ventilation; ICU, intensive care unit.

^a^ Values are expressed as mean ± SD or No. (%).

^b^ P < 0.05 was considered statistically significant.

## 5. Discussion

Sedation is often administered to patients on MV to improve tolerance of the endotracheal tube and facilitate adaptation to the ventilator. Sedatives such as dexmedetomidine and propofol are commonly used to reduce the stress response, anxiety, and pain associated with MV (22). Multiple studies have explored the use of dexmedetomidine and propofol for these purposes (4, 6, 23, 24). 

In this study, SAA levels, a recognized stress biomarker, were used to assess the stress-mitigating effects of these sedatives. While no significant difference in SAA levels was observed immediately after MV initiation, group D (dexmedetomidine) showed significantly lower SAA levels at 12 hours (66.88 ± 8.9 vs. 72.14 ± 6.62, P = 0.041), 24 hours (63.8 ± 7.07 vs. 68.36 ± 5.99, P = 0.034), 36 hours (59.36 ± 7.35 vs. 66.29 ± 6.03, P = 0.002), and 48 hours (52.41 ± 7.33 vs. 61.48 ± 6.19, P < 0.001) compared to group P (propofol). This suggests that dexmedetomidine, an alpha-2 adrenergic receptor agonist with high selectivity, exerts a more potent stress-reducing effect in MV patients by modulating catecholamine release and, consequently, SAA levels, without causing respiratory depression (8, 16, 18, 25-27). These results align with previous studies that found lower SAA levels in dexmedetomidine-treated hypertensive patients undergoing elective surgery (19).

Hemodynamic evaluation showed no significant differences in HR and MAP immediately after MV initiation between the two groups. However, at 12, 18, and 24 hours, group D exhibited significantly lower HR and MAP compared to group P, indicating that dexmedetomidine provided better hemodynamic stability. These findings are consistent with previous studies demonstrating dexmedetomidine's ability to stabilize hemodynamics through its alpha-2 agonist properties (4, 19, 28), although some studies have reported differing results (23). This further highlights dexmedetomidine's potential in maintaining hemodynamic stability, especially in critically ill patients.

Importantly, the duration of MV in group D was significantly shorter (4.4 ± 1.85 days) compared to group P (6.1 ± 2.45 days, P = 0.018). This finding is consistent with several studies that have also reported a reduced MV duration with dexmedetomidine (5, 13), although some inconsistencies exist in the literature (24). 

No significant differences were observed in secondary outcomes, including ICU length of stay, ICU mortality, bradycardia, or hypotension, which aligns with some prior studies (23, 24). While there was a trend toward a shorter ICU stay in the dexmedetomidine group (6.85 ± 1.27 days vs. 7.8 ± 1.94 days, P = 0.074), it did not reach statistical significance. This could be due to various factors, such as the complex determinants of ICU stay, including illness severity and comorbidities, as well as the study's relatively small sample size, which may have limited its power. Additionally, the short overall ICU stays in both groups may suggest a ceiling effect, reducing the impact of the sedation strategy. Regarding ICU mortality (10% in the dexmedetomidine group vs. 20% in the propofol group, P = 0.661), the lack of significant differences may be attributable to the limited study power, patient population heterogeneity, and short follow-up period. The rates of bradycardia (20% vs. 10%, P = 0.661) and hypotension (35% vs. 20%, P = 0.480) were also similar between the two groups, potentially reflecting careful titration of both sedatives and adherence to standardized ICU management protocols.

The study's limitations include the relatively small sample size, the use of a single-center design, lack of blinding, and potential biases inherent in an open-label trial. Additionally, the investigation did not assess long-term clinical outcomes, such as overall hospitalization duration or long-term mortality. Moreover, other biomarkers were not evaluated in relation to SAA levels, which could provide further insights into the physiological response to sedation.

### 5.1. Conclusions

In conclusion, our open-label randomized trial demonstrated the superior stress-mitigating effects of dexmedetomidine compared to propofol in MV patients, as indicated by reduced SAA levels and improved hemodynamic stability. These findings, alongside a significant reduction in the duration of MV, suggest potential clinical advantages for dexmedetomidine in this patient population. However, to overcome the limitations of this study, further research is essential. Future investigations should focus on expanding sample sizes, extending follow-up periods, incorporating blinding protocols, and including more diverse patient populations to enhance the generalizability of the results. Additionally, exploring alternative methodologies or additional variables may offer deeper insights into the clinical and physiological effects of sedatives in critically ill patients.

## Data Availability

The dataset presented in the study is available on request from the corresponding author during submission or after publication.

## Clinical Trial Registration Code:

NCT06098209.
